# Mechanical signals control *SOX-9*, *VEGF*, and *c-Myc *expression and cell proliferation during inflammation via integrin-linked kinase, B-Raf, and ERK1/2-dependent signaling in articular chondrocytes

**DOI:** 10.1186/ar3039

**Published:** 2010-05-28

**Authors:** Priyangi M Perera, Ewa Wypasek, Shashi Madhavan, Birgit Rath-Deschner, Jie Liu, Jin Nam, Bjoern Rath, Yan Huang, James Deschner, Nicholas Piesco, Chuanyue Wu, Sudha Agarwal

**Affiliations:** 1 Biomechanics and Tissue Engineering Laboratory, The Ohio State University, Postle Hall, 305 W 12th Avenue, Columbus, OH 43210, USA; 2 Department of Orthodontics, University of Bonn, Welschnonnenstrasse 17, 53111 Bonn, Germany; 3 Department of Orthopedics, University of Regensburg, Kaiser-Karl V-Allee 3, 93077 Bad Abbach, Germany; 4 Department of Periodontics, University of Bonn, Welschnonnenstrasse 17, 53111 Bonn, Germany; 5 Department of Oral Biology, School of Dental Medicine, Salk Hall, 3501 Terrace Street, University of Pittsburgh, Pittsburgh, PA 15261, USA; 6 Department of Pathology, University of Pittsburgh, School of Medicine, S-417 BST, 200 Lothrop Street, Pittsburgh, PA 15261, USA

## Abstract

**Introduction:**

The importance of mechanical signals in normal and inflamed cartilage is well established. Chondrocytes respond to changes in the levels of proinflammatory cytokines and mechanical signals during inflammation. Cytokines like interleukin (IL)-1β suppress homeostatic mechanisms and inhibit cartilage repair and cell proliferation. However, matrix synthesis and chondrocyte (AC) proliferation are upregulated by the physiological levels of mechanical forces. In this study, we investigated intracellular mechanisms underlying reparative actions of mechanical signals during inflammation.

**Methods:**

ACs isolated from articular cartilage were exposed to low/physiologic levels of dynamic strain in the presence of IL-1β. The cell extracts were probed for differential activation/inhibition of the extracellular signal-regulated kinase 1/2 (ERK1/2) signaling cascade. The regulation of gene transcription was examined by real-time polymerase chain reaction.

**Results:**

Mechanoactivation, but not IL-1β treatment, of ACs initiated integrin-linked kinase activation. Mechanical signals induced activation and subsequent C-Raf-mediated activation of MAP kinases (MEK1/2). However, IL-1β activated B-Raf kinase activity. Dynamic strain did not induce B-Raf activation but instead inhibited IL-1β-induced B-Raf activation. Both mechanical signals and IL-1β induced ERK1/2 phosphorylation but discrete gene expression. ERK1/2 activation by mechanical forces induced SRY-related protein-9 (SOX-9), vascular endothelial cell growth factor (VEGF), and c-Myc mRNA expression and AC proliferation. However, IL-1β did not induce SOX-9, VEGF, and c-Myc gene expression and inhibited AC cell proliferation. More importantly, SOX-9, VEGF, and Myc gene transcription and AC proliferation induced by mechanical signals were sustained in the presence of IL-1β.

**Conclusions:**

The findings suggest that mechanical signals may sustain their effects in proinflammatory environments by regulating key molecules in the MAP kinase signaling cascade. Furthermore, the findings point to the potential of mechanosignaling in cartilage repair during inflammation.

## Introduction

Mechanical loading during joint movement is critical for cartilage function and survival. Chondrocytes located within the cartilage recurrently experience mechanical forces during joint movements. These cells sense, interpret, and respond to mechanical signals to maintain tissue integrity and homeostasis [1-5]. Activation of cells by mechanical signals is a rapid process and leads to activation of several intracellular signaling cascades, flow channels, and genes [6-8]. Accumulating evidence suggests that chondrocytic mechanosensing is discriminatory and capable of recognizing and responding to signals of various magnitudes to differentially regulate cartilage repair and pathologies [4,9].

Similarly to soluble ligands, mechanotransduction is initiated at the matrix-membrane interface [10,11]. Chondrocytes located in the extracellular matrix are believed to relay mechanical signals through the plasma membrane via integrins [12,13]. Integrin-linked kinase (ILK), located in the cytoplasmic domain of integrins, plays a key role in transmitting mechanical signals to the intracellular compartment [13-15]. Within the cells, Ras (p21), Rho, and Rac belonging to the GTPase family of proteins are stimulated following activation of ILK and certain growth factor receptors [16,17]. Ras activation via exchange of guanosine diphosphate (GDP) to guanosine triphosphate (GTP) allows Ras to bind proto-oncogene c-RAF kinases (Rafs) via Ser/Thr/Tyr phosphorylation of A-Raf, B-Raf, and c-Raf at multiple sites [18]. Phosphorylated Rafs activate mitogen-activated protein kinase (MAPK) kinase (MEK1/2) by phosphorylation of Ser217/Ser221 [19]. Subsequently, MEK1/2 activates extracellular receptor kinase 1/2 (ERK1/2) by phosphorylating Thr202/Tyr204. ERK1/2 activation is associated with growth signals. However, cytokines like interleukin-1 (IL-1) and tumor necrosis factor-alpha (TNF-α) also phosphorylate ERK1/2 to regulate certain proinflammatory genes [20,21]. Following activation, ERK1/2 translocates to the nucleus and activates transcription factors that are specific to the signals perceived by cells [22].

During inflammation, chondrocytes are exposed to proinflammatory cytokines such as IL-1β and TNF-α. These cytokines alter their chondrogenic potential, prevent cell proliferation, and induce dedifferentiation and apoptosis. Specifically, cells exposed to IL-1β lose their ability to express SRY-related protein-9 (SOX-9) and vascular endothelial cell growth factor (VEGF) [23]. However, mechanical signals are shown to be reparative and upregulate proliferation and expression of collagen type II and proteoglycans in articular chondrocytes (ACs). These signals activate ERK1/2, suggesting a role for this signaling cascade in cartilage repair [12,24]. In this study, we investigated the intracellular signaling events responsible for beneficial/reparative effects of mechanical signals during inflammation. We demonstrate that mechanical signals and IL-1β both regulate the ERK1/2 signaling cascade but lead to activation of disparate transcription factors and gene expression. Strikingly, the actions of mechanical signals are sustained in the inflammatory environment and upregulate SOX-9, VEGF, and c-Myc gene transcription as well as chondrocyte proliferation.

## Materials and methods

### Cell isolation, culture, and exposure to dynamic tensile or compressive forces

ACs were isolated from knee joints of 12- to 14-week-old, female, Sprague Dawley rats (Charles River Laboratories, Inc., Wilmington, MA, USA) as described earlier. Briefly, cartilage from the condyles of femurs and tibia were aseptically removed, chipped, and digested in 1,400 U/mL collagenase type I (Worthington Biochemical Corporation, Lakewood, NJ, USA) for 3 hours at 37°C. The cells were washed and grown in medium (tissue culture medium, or TCM) containing Ham's F12, 10% fetal bovine serum (FBS), 10 U penicillin, 10 μg/mL streptomycin, and 2 mM glutamine (Invitrogen Corporation, Carlsbad, CA, USA). Cells were used in the first three passages.

ACs were subjected to dynamic tensile forces (dynamic strain, or DS) as described previously [3,25]. Briefly, ACs (6 × 10^4^/3 mL TCM per well) were plated in Bioflex plates (Flexcell International Corporation, Hillsborough, NC, USA) and cultured for 5 days to attain 70% to 80% confluence. Subsequently, 18 hours prior to exposing cells to DS or IL-1β, the medium was replaced with TCM containing 1% FBS. Cells were exposed to DS at a magnitude of 6% and 0.25 Hz for the required time interval and the mRNA or proteins were extracted as described below.

### Western blot analysis

Western blot assays were performed as described previously. Briefly, AC cells were lysed in Ripa buffer (Santa Cruz Biotechnology, Inc., Santa Cruz, CA, USA) containing protease and phosphatase inhibitor cocktail-2 (Sigma-Aldrich, St. Louis, MO, USA). The cell lysates were subjected to SDS-10%-PAGE, electrotransferred to a nitrocellulose membrane (Bio-Rad Laboratories, Inc., Hercules, CA, USA), and reacted with antibodies to phospho-Thr202/Tyr204 ERK1/2 and total ERK1/2, phospho-Ser 217/221 MEK1/2 and total MEK1/2, phospho-Ser338 cRaf, phospho-Ser445 B-Raf, phospho-Thr423-PAK1, phospho-Thr58/Ser62 Myc, and total c-Myc proteins (Cell Signaling Technology, Inc., Danvers, MA, USA). Protein loading was normalized with total β-actin or antibodies to total signaling molecule in each sample. The primary antibodies were probed with horseradish peroxidase (HRP)- or IR-Dye 680- or IR-Dye-880-conjugated secondary antibodies and scanned using a Kodak 1000 Image Documentation System (Eastman Kodak Company, Rochester, NY, USA) for HRP or an Odyssey infrared imaging system (LI-COR Biosciences, Lincoln, NE, USA) for IR-Dye-labeled antibodies. In some experiments, cells were pretreated with various inhibitors such as ERK inhibitor PD98059 (SABiosciences Corporation, Frederick, MD, USA) or Ras inhibitor GGT12133 (Pierce, Rockford, IL, USA) at the specified concentrations 30 minutes prior to mechanoactivation or IL-1 treatment or both.

### RAS activation

The activated RAS in cells was estimated with an Active Ras Pull-Down and Detection Kit (Pierce) in accordance with the manufacturer's recommended protocol. Briefly, glutathione-S-transferase (GST) fusion protein containing the Ras-binding domain (RBD) of Raf1 (GST-Raf1-RBD; approximately 42 kDa) was incubated with cell lysate and glutathione agarose beads. The active Ras bound to the GST-Raf1-RBD was pulled down by centrifugation, and active RAS was detected by Western blot analysis using anti-Ras antibody. Control reactions using GTPγ and GDP were performed to ensure that only active RAS was bound to GTP.

### Real-time polymerase chain reaction

Total RNA was extracted with an RNeasy Micro Kit (Qiagen Inc., Valencia, CA, USA), and real-time polymerase chain reaction (RT-PCR) was conducted as described earlier [3]. Gene-specific primers used to amplify the cDNA (SYBR Green Master Mix; Bio-Rad Laboratories, Inc.) were rat VEGF (sense) GCCTTGTTCAGAGCGGAGAAA and (anti-sense) CGCGAGTCTGTGTTTTTGCA, rat MYC (sense) GGAAAACAACGAAAAGGCCC and (antisense) TGCTCATCTGCTTGAACGGAC, and rat SOX-9 (sense) ATCTGAAGAAGGAGAGCGAG and (antisense) CAAGCTCTGGAGACTGCTGA. Collected data were analyzed by the comparative threshold cycle method [26].

### Cell proliferation assay

The cell proliferation was examined over a 3-day period by the MTT (3-(4,5 dimethylthiazolyl-2)-2,5-diphenyltetrazolium bromide) cell proliferation assay (American Type Culture Collection, Manassas, VA, USA) in accordance with the manufacturer's recommended protocol. The cells following treatment were incubated for 3 hours with 100 μL/mL MTT (5 mg/mL Hanks' balanced salt solution), and the formazan formation was assessed by absorbance at 450 nm (Victor Plate Reader; PerkinElmer Inc., Waltham, MA, USA). The cell proliferation was calculated as mean absorbance of cells exposed to DS divided by mean absorbance of controls.

### Transfection of ACs with wild-type and mutant forms of FLAG-tagged ILK

To examine the role of ILK in ERK1/2 activation, ACs were transfected with FLAG (polypeptide protein sequence DYKDDDDK)-ILK expression vectors, which were kindly provided by Chuanyue Wu, of the University of Pittsburgh (Pittsburgh, PA, USA). ACs grown to 70% confluence were transfected with various expression plasmids containing wild-type (WT) ILK cDNA (residues 1 to 145; pFLAG-WT-ILK), the kinase-deficient (KD) ILK mutant containing a single mutation at Glu359 for Lys (pFLAG-KD-ILK), the N-terminal deletion (residues 1 to 230; pFLAG-N-ILK), or the mock transfectants pFLAGCMV-2 [15], using Lipofectamine 2000 (Invitrogen Corporation) as specified by the manufacturer. Expression of FLAG-ILK proteins was confirmed by immunofluorescence staining with a mouse monoclonal anti-FLAG antibody (Sigma-Aldrich). After transfection for 24 hours, the cells were fed with fresh selective medium containing G418 geneticin (800 μg/mL; Invitrogen Corporation). Neomycin-resistant clones were cultured in selective medium for another passage and then transferred into Bioflex II six-well plates for experimentation.

### Immunofluorescence staining of ACs

Immunofluorescence staining was performed as described earlier [27]. Briefly, cells were fixed with 2% paraformaldehyde, permeabilized with 0.2% Triton × 100 in phosphate-buffered saline, and washed and stained with primary antibodies followed by CY3-labeled secondary antibodies. Beta-actin was stained with fluorescein isothiocyanate-labeled phalloidin (Sigma-Aldrich).

## Results

### Mechanical signals induce AC proliferation in the absence or presence of IL-1β

**Figure 1:**
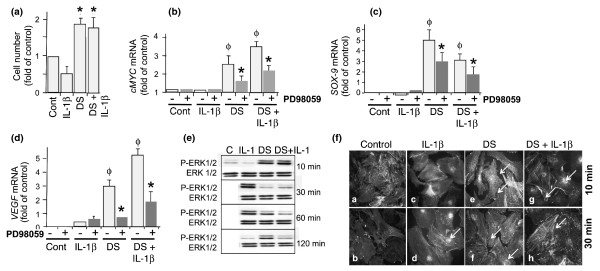
**Mechanical signals upregulate articular chondrocyte (AC) proliferation via SOX-9, VEGF, and c-Myc mRNA expression and ERK1/2 activation**. ACs were exposed to no treatment or to treatment with interleukin-1-beta (IL-1β), dynamic strain (DS) alone, or DS and IL-1β. Subsequently, ACs were subjected to DS for 90 minutes per day for 3 days. **(a) **On day 4, the rate of cell proliferation was assessed by MTT assay. ACs were treated either with medium alone or with PD98059 (2 μM) for 30 minutes. Cells were exposed to the treatment regimens above for 3 hours, and the mRNA expression for *c-Myc ***(b)**, *SOX-9 ***(c)**, and *VEGF ***(d) **was analyzed by real-time polymerase chain reaction. **(e) **Western blot analysis showing ERK1/2 phosphorylation using phospho-Thr202/Tyr204 ERK1/2 (P-ERK1/2) and total ERK1/2 (T-ERK1/2) antibodies. **(f) **Immunofluorescence analysis showing minimal phospho-ERK1/2 in control cells [a], cells stained with secondary antibody alone [b], optimal phosphorylation of ERK1/2 and its nuclear translocation in response to IL-1β at 10 and 30 minutes [c,d], and nuclear translocation and cytoplasmic redistribution of p-ERK1/2 in response to DS in the absence [e,f] and presence [g,h] of IL-1β. Cells were counterstained with fluorescein isothiocyanate-phalloidin to show β-actin. Experiments in (a,c-e) were performed in triplicate and were repeated three times in (b) and two times in (f). The error bars represent standard error of the mean (standard error of the mean in a-d). Gels in (e) represent one of three experiments with similar results. ^Φ ^*P  *< 0.05 as compared with untreated controls; *P  < 0.05 as compared with cells treated with DS or with DS and IL-1. C, control; Cont, control; ERK1/2, extracellular receptor kinase 1/2; MTT, 3-(4,5 dimethylthiazolyl-2)-2,5-diphenyltetrazolium bromide; SOX-9, SRY-related protein-9; VEGF, vascular endothelial cell growth factor.

To gain insight into the actions of mechanical signals during inflammation, we first determined AC proliferation in the presence of IL-1β. ACs grown on Bioflex plates were mechanoactivated for 90 minutes per day for 2 days with medium alone or medium containing IL-1β. On day 3, spectrophotometric determination of cells by MTT assay revealed that exposure of ACs to mechanical signals significantly upregulated cell proliferation. However, IL-1β significantly suppressed AC proliferation (Figure 1a).

### Mechanoactivation of ACs leads to c-Myc, VEGF, and SOX-9 mRNA expression

VEGF, c-Myc, and SOX-9 are all involved in AC proliferation and differentiation. Therefore, we next determined whether mRNA expression for c-Myc, VEGF, and SOX-9 is upregulated in mechanoactivated ACs in the absence or presence of IL-1β. RT-PCR analysis showed that mechanoactivation of ACs significantly upregulated c-Myc, SOX-9, and VEGF mRNA expression involved in AC proliferation and differentiation (Figure 1b-d). We next examined whether ERK1/2 activation was required for the upregulation of mRNA expression for these genes. ACs pretreated for 30 minutes with PD98059 (a MEK1/2- and ERK1/2-specific inhibitor) and then exposed to DS showed a significant suppression of DS-induced mRNA expression for c-Myc, SOX-9, and VEGF (Figure 1b-d). IL-1β did not induce expression of c-Myc, SOX-9, or VEGF significantly. However, PD98059 significantly abolished DS-dependent c-Myc, SOX-9, and VEGF mRNA induction in the presence of IL-1β. These findings suggested that DS induces VEGF and SOX-9 mRNA expression via the ERK1/2 signaling cascade.

### Mechanical signals activate ERK1/2 in the absence or presence of IL-1β

Since DS-induced VEGF and SOX-9 were inhibited by PD98059, we next confirmed whether mechanical signals induced ERK1/2 activation. DS significantly upregulated Thr202/Tyr204-ERK1/2 phosphorylation within 10 minutes and was dephosphorylated in the ensuing 20 minutes (Figure 1e). Thereafter, ERK1/2 reactivation was observed at 60 and 120 minutes. In cells treated with IL-1β, phosphorylation of ERK1/2 was delayed but sustained between 30 and 60 minutes. More importantly, in cells simultaneously exposed to IL-1β and DS, ERK1/2 was activated within 10 minutes and was subsequently dephosphorylated by 30 minutes. Immunofluorescence staining of ACs revealed that the phosphorylation of ERK1/2 was paralleled by its nuclear translocation and cytoplasmic redistribution in cells treated with DS or with DS and IL-1β (Figure 1f). In cells treated with IL-1β, the majority of phospho-ERK1/2 was located in the nuclei at 30 minutes (Figure 1f).

### Mechanical signals suppress IL-1β-induced B-Raf activation

**Figure 2:**
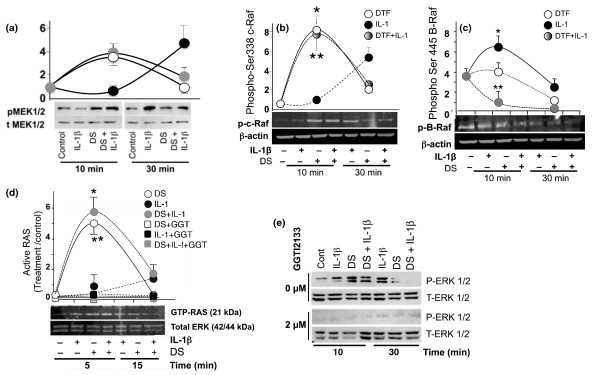
**Mitogen-activated protein kinase signaling in response to dynamic strain (DS)**. Articular chondrocytes (ACs) were exposed to no treatment or to treatment with interleukin-1-beta (IL-1β), DS alone, or DS and IL-1β for 10 or 30 minutes and were examined by Western blot analysis for **(a) **Ser217/221-MEK1/2 phosphorylation by DS and IL-1β. Equal protein loading was confirmed by probing blots with anti-total MEK1/2 antibody. **(b) **Ser338-c-RAF phosphorylation, **(c) **Ser445-B-Raf phosphorylation, and **(d) **Ras activation following immunoprecipitation with GST-Raf-1-RBD and glutathione agarose beads are shown. **(e) **Ras activation following pretreatment of cells with a selective Ras antagonist (2 μM GGT12133) for 30 minutes is shown along with the assessment of Ras-dependent phospho-Thr202/Tyr204-ERK1/2 (P-ERK1/2) 10 and 30 minutes post-activation. Anti-total ERK1/2 IgG (T-ERK1/2) was used to normalize total input in all lanes. All experiments were performed in triplicate. Gels represent one of three experiments with similar results in (a-e). The graphs above gels in each figure show mean and standard error of the mean of phosphoprotein/total protein in three separate experiments in (A-D). *P  < 0.05 as compared with untreated control cells; **P  < 0.05 as compared with IL-1β-treated cells. DTF, dynamic tensile force; ERK1/2, extracellular receptor kinase 1/2; GGT, Ras inhibitor GGT12133; GST, glutathione-S-transferase; MEK1/2, mitogen-activated protein kinase/extracellular receptor kinase 1/2; RBD, Ras-binding domain.

To understand how mechanical signals sustain their effects in the presence of IL-1β, we examined the events upstream of ERK1/2. Western blot analysis using anti-phospho-Ser 217/221 MEK1/2 and total MEK1/2 showed that DS induced a rapid and transient phosphorylation of MEK1/2 within 10 minutes. IL-1-induced MEK1/2 activation was observed after 30 minutes of cell activation. Similarly to DS alone, mechanoactivation of cells in the presence of IL-1β showed a rapid and transient phosphorylation of MEK1/2 within 10 minutes (Figure 2a).

Since phosphorylation of Raf kinases is necessary for MEK1/2 activation, we next determined whether A-Raf, B-Raf, or c-Raf is activated by DS. DS or IL-1β did not activate A-Raf (data not shown). DS alone or in the presence of IL-1β induced a rapid phosphorylation of Ser338 on c-Raf (Figure 2b). B-Raf was constitutively phosphorylated in ACs. Western blot analysis demonstrated that IL-1β significantly activated B-Raf by phosphorylating its Ser445 residues. However, B-Raf was not activated by DS but it did suppress IL-1β-induced Ser445-B-Raf phosphorylation (Figure 2c).

Using a similar experimental strategy, we next examined the activation of the RAS proteins. RAS proteins are found as GTP-bound active and GDP-bound inactive forms. ACs exposed to the above experimental regimens were lysed and subjected to precipitation to capture activated RAS with GST-Raf-RBD and glutathione agarose beads. Western blot analysis revealed that DS alone or in the presence of IL-1β induced a rapid but transient activation of RAS within 5 minutes (Figure 2d). However, IL-1β induced a minimal RAS activation. Untreated ACs exhibited negligible GTP-bound activated RAS. To confirm these observations, ACs were further pretreated with a selective antagonist of RAS, GGT12133 (2 or 10 μM), and subsequently stimulated for 5 or 15 minutes. GGT12133 (2 μM) completely inhibited DS-induced ERK1/2 activation, confirming that mechanical signals induce RAS activation in the absence or presence of an inflammatory stimulus (Figure 2e).

### Mechanical signals activate ILK to initiate ERK1/2 signaling cascade

**Figure 3:**
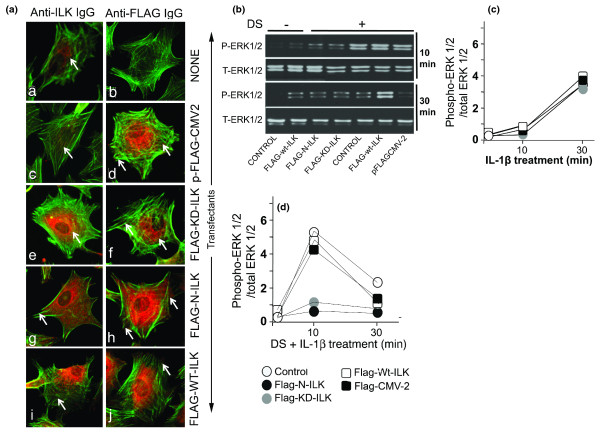
**Mechanical signals activate integrin-linked kinase (ILK)**. **(a) **Articular chondrocytes (ACs) either were not transfected [a,b] or were transfected with p-FLAG-CMV2 empty [c,d], FLAG-KD-ILK [e,f], FLAG-N-ILK [g,h], or FLAG-WT ILK [i,j]. ACs were immunostained with anti-ILK (left frames) or anti-FLAG (right frames) antibodies and CY3-conjugated secondary antibodies. All cells were counterstained with fluorescein isothiocyanate-phalloidin to visualize β-actin. Western blot analysis shows ERK1/2 activation in untransfected ACs or those transfected with FLAG-N-ILK, FLAG-KD-ILK, FLAG-WT-ILK, or pFLAG-CMV2 exposed to **(b) **no strain or dynamic strain (DS) alone, **(c) **interleukin-1-beta (IL-1β) alone, or **(d) **DS and IL-1β. Frames (c,d) show semiquantitative estimation of bands in Western blots. All figures represent one of three similar experiments. ERK1/2, extracellular receptor kinase 1/2; FLAG, polypeptide protein sequence DYKDDDDK; KD, kinase-deficient; P-ERK1/2, phospho-Thr202/Tyr204-extracellular receptor kinase 1/2; T-ERK1/2, total extracellular receptor kinase 1/2; WT, wild-type.

ILK is shown to activate RAS proteins. To determine whether ILK activation was necessary for mechanoactivation-induced RAS activation, ACs were transfected with plasmids containing FLAG-ILK expression vectors containing the full-length ILK (FLAG-WT-ILK), truncated N terminal (residues 1-230, FLAG-N-ILK), and the KD ILK mutant (FLAG-KD-ILK) containing a single mutation (Glu359 at Lys) or with pFLAG-CMV-2 vector lacking the ILK sequence as a control [28,29]. ACs shown in Figure 3a were untransfected (a,b) or were transfected with FLAG-CMV-2 empty vector (c,d), FLAG-KD-ILK (e,f), mutant FLAG-N-ILK (g,h), or FLAG-WT-ILK (i,j), and ILK was detected by rabbit anti-ILK (a,c,e,g,i) or rabbit anti-FLAG antibodies (b,d,f,h,j). Cells stained with goat anti-rabbit CY3-labeled secondary antibodies alone did not show staining (data not shown).

Western blot analysis showed that untransfected control cells and those transfected with FLAG-WT-ILK did not exhibit constitutive ERK1/2 phosphorylation (Figure 3b). However, within 10 minutes, exposure of untransfected control cells and cells transfected with pFLAG-CMV-2 or FLAG-WT-ILK to DS showed ERK1/2 phosphorylation, which remained high in cells overexpressing WT-ILK. However, mechanoactivation of ACs transfected with FLAG-N-ILK or FLAG-KD-ILK failed to induce ERK1/2 phosphorylation in cells (Figure 3b). Densitometric analysis of the same samples probed with anti-total ERK1/2 antibody confirmed equal protein input in all lanes (Figure 3b). ACs activated by IL-1β showed ERK1/2 activation in cells transfected with FLAG-mutant-ILK or FLAG-WT-ILK following 30 minutes of activation (Figure 3c). However, cells simultaneously activated with IL-1β and DS showed ERK1/2 activation in only the untransfected cells or those transfected with plasmids containing FLAG-WT-ILK or pFLAG-CMV-2 (Figure 3d).

## Discussion

We have shown that dynamic mechanical signals vitally control AC proliferation and differentiation by regulating the MAPK signaling cascade. Furthermore, the actions of mechanical signals are sustained in the presence of proinflammatory signals induced by IL-1β. We have exposed ACs to dynamic tensile forces to assess their potential in controlling cell growth. During joint movement, ACs simultaneously experience dynamic compression-, tension-, and torsion-induced forces. *In vitro*, ACs subjected to 10% compression in three-dimensional microfiber or agarose constructs exhibit many biochemical changes similar to those of ACs exposed to 6% tensile forces. For example, 10% compressive forces as well as 6% tensile forces suppress proinflammatory gene induction, upregulate total proteoglycan contents, and aggrecan, collagen type II, and SOX-9 mRNA induction in ACs [7,8,30-32]. Therefore, in this study, 6% tensile forces were used to examine the signaling events induced by DS. However, so far, the extent of compressive or tensile forces experienced by ACs during joint movement *in vivo *is not clear.

Intracellular signal transduction by mechanical signals begins with ILK activation. This was evident by the observations that mechanical signals failed to induce ERK1/2 phosphorylation in ACs transfected with mutant-ILK or kinase activity-deficient ILK plasmids. However, mechanical signals induced ERK1/2 activation in ACs transfected with WT ILK or untransfected cells. These studies revealed that ILK activation by mechanical signals is of critical importance given the fact that integrins are the putative mechanosensors of chondrocytes, and ILK is one of the central signaling components of the integrin complex [15]. Interestingly, mechanical signals are also perceived via integrins to activate Rho GTPases to regulate cytoskeletal rearrangements [33]. This indicates that mechanical signals regulate diverse cellular functions via integrin engagement.

Mechanoactivation of ACs leads to the rapid activation of RAS. In an effort to examine whether mechanical signals regulate RAS during inflammation, we examined the effects of IL-1β on RAS activation. IL-1β induces minimal activation of RAS. Nevertheless, RAS activation is similar in mechanoactivated cells irrespectively of the presence of IL-1β. RAS activation is associated with ERK1/2-mediated cell proliferation [34]. Consistent with these findings, our data show that the RAS inhibitor GGT12133 attenuates ERK1/2 phosphorylation induced by mechanical signals. RAS activation is central to activation of many cell surface receptors, such as growth factor receptors, receptor tyrosine kinases, integrins, and IL-6 receptors [34-36], further suggesting that dynamic mechanical signals activate signaling molecules similar to other growth factors.

To examine how mechanical signals and IL-1β regulate ERK1/2 signaling cascade that result in differential gene expression, we next examined the activation of Rafs [37]. Mechanical signals trigger c-Raf kinase activity by phosphorylating Ser338 residues. However, IL-1β induces Ser445-B-Raf phosphorylation. B-Raf was not activated by mechanical signals. However, mechanical signals inhibited IL-1β-induced B-Raf activation. This disparity in the activation of Rafs may play a critical role in the differential processing of signals generated by IL-1β and mechanical forces. However, the mechanisms that underlie this regulation of c-Raf and B-Raf remain to be elucidated.

Activation of B-Raf by IL-1β or c-Raf by mechanical signals results in MEK1/2 activation via Ser217/221 phosphorylation [19]. Subsequently, MEK1/2 activates ERK1/2 by phosphorylating both Thr202/Tyr204 residues. Following mechanoactivation, phosphorylated ERK1/2 rapidly translocates to the nucleus and is redistributed to the cell surface. ERK proteins after activation translocate to the nuclear compartment, where they act as the main executor of ERK1/2 biological functions, and channel a diverse array of signals via downstream targets. Additionally, ERK dimers and scaffolds translocate to cognate cytoplasmic substrates, where they stabilize ERK1/2 and Myc functions in cell proliferation [35,38,39].

Interestingly, ERK1/2 activation is temporally regulated in response to DS as well as IL-1β. DS rapidly induces ERK1/2 phosphorylation, which is observed within 10 minutes. IL-1β-induced ERK1/2 phosphorylation is apparent at 30 minutes. It is likely that DS, by activating kinases upstream of ERK1/2, initiates a feedback loop that suppresses IL-1β-induced ERK1/2 activation. Such early activation of ERK1/2 by DS may likely play a role in sustaining its effects in the presence of IL-1β.

Mechanoactivation of ACs leads to c-Myc, VEGF, and SOX-9 mRNA expressions, all of which have been implicated in the proliferative response of cells to a variety of stimuli [35,40]. Furthermore, ERK1/2 activation is required for c-Myc, SOX-9, and VEGF mRNA expression, as evidenced by the suppression of their transcriptional activation by PD98059. We have also observed that ERK1/2 activation by IL-1β fails to induce SOX-9 or VEGF expression. This may explain the suppression of AC proliferation in the presence IL-1β. These findings again point to similarities between mechanical signals and other growth factors that use the ERK1/2/Myc signaling cascade to regulate cell proliferation [23,36,41]. Furthermore, the fact that mechanical signals upregulate c-Myc, SOX-9, and VEGF in the presence of IL-1β supports the benefits of mechanoactivation of ACs in the inflamed cartilage.

## Conclusions

Our findings demonstrate for the first time that mechanical signals suppress the ERK1/2 signaling cascade of IL-1β, indicating a critical role for these signals in rescuing cartilage from the detrimental effects of IL-1β during inflammation. The cellular decision-making in response to mechanical forces occurs swiftly and is phospho-relayed via ILK to downstream signaling targets. Nonetheless, activation of intermediate signaling molecules like c-Raf and B-Raf may be critical in regulating ERK1/2 transcriptional activity in response to mechanosignaling. Only c-Raf is activated by mechanical signals but it inhibits B-Raf activation by IL-1β. Activated hetrodimers and homodimers of B-Raf and c-Raf regulate downstream activation of MAPKs. By suppressing B-Raf activation, mechanical signals may likely alter a critical event important for the downstream IL-1β signaling. This may lead to the SOX-9, VEGF, and Myc upregulation responsible for cell proliferation in IL-1β-treated cells. Earlier studies have shown that mechanical signals also suppress inflammation by inhibiting nuclear factor-kappa-B activation and thus expression of proinflammatory genes, such as IL-1β, TNF-α, inducible nitric oxide synthase, matrix metalloproteinases, and lipopolysaccharide [3,7,8,25]. The present findings thus demonstrate, at least in part, the basis for the regenerative potential of mechanical signals in arthritic diseases. Furthermore, studies show the importance of the ERK1/2 signaling cascade in mediating proliferative actions of mechanical signals in proinflammatory environments.

## Abbreviations

AC: articular chondrocyte; DS: dynamic strain; ERK1/2: extracellular receptor kinase 1/2; FBS: fetal bovine serum; FLAG: polypeptide protein sequence DYKDDDDK; GDP: guanosine diphosphate; GST: glutathione-S-transferase; GTP: guanosine triphosphate; HRP: horseradish peroxidase; IL-1: interleukin-1; ILK: integrin-linked kinase; KD: kinase-deficient; MAPK: mitogen-activated protein kinase; MEK1/2: mitogen-activated protein kinase/extracellular receptor kinase 1/2; MTT: 3-(4,5 dimethylthiazolyl-2)-2,5-diphenyltetrazolium bromide; RAF: proto-oncogene c-RAF kinase; RBD: Ras-binding domain; RT-PCR: real-time polymerase chain reaction; SOX-9: SRY-related protein-9; TCM: tissue culture medium; TNF-α: tumor necrosis factor-alpha; VEGF: vascular endothelial cell growth factor; WT: wild-type.

## Competing interests

The authors declare that they have no competing interests.

## Authors' contributions

PMP carried out Ras activation, AC isolation, DS exposure, and SOX-9, VEGF, and Myc induction. EW performed ERK and MAPK activation. SM carried out Raf/Ras activation. BR-D carried out activation and expression of ERK-responsive transcription factors. JL was responsible for immunofluorescence. JN performed stretch protocol optimization. BR carried out ILK activation. YH was responsible for activation of transcription factors. JD conducted experimental planning and data management. NP prepared the manuscript. CW prepared ILK constructs. SA conducted experimental planning and prepared the manuscript. All authors read and approved the final manuscript.
